# Glial Fibrillary Acidic Protein-Expressing Glia in the Mouse Lung

**DOI:** 10.1177/1759091415601636

**Published:** 2015-10-06

**Authors:** Gabriela B. Suarez-Mier, Marion S. Buckwalter

**Affiliations:** 1 Department of Neurology and Neurological Sciences, Stanford Medical School, Stanford, CA, USA; 2 Stanford Neurosciences Institute, Stanford, CA, USA; 3 Department of Neurosurgery, Stanford Medical School, Stanford, CA, USA

**Keywords:** glia, Remak, pulmonary innervation, autonomic, glial fibrillary acidic protein-Cre, neuroepithelial body

## Abstract

Autonomic nerves regulate important functions in visceral organs, including the lung. The postganglionic portion of these nerves is ensheathed by glial cells known as non-myelinating Schwann cells. In the brain, glia play important functional roles in neurotransmission, neuroinflammation, and maintenance of the blood brain barrier. Similarly, enteric glia are now known to have analogous roles in gastrointestinal neurotransmission, inflammatory response, and barrier formation. In contrast to this, very little is known about the function of glia in other visceral organs. Like the gut, the lung forms a barrier between airborne pathogens and the bloodstream, and autonomic lung innervation is known to affect pulmonary inflammation and lung function. Lung glia are described as non-myelinating Schwann cells but their function is not known, and indeed no transgenic tools have been validated to study them *in vivo*. The primary goal of this research was, therefore, to investigate the relationship between non-myelinating Schwann cells and pulmonary nerves in the airways and vasculature and to validate existing transgenic mouse tools that would be useful for studying their function. We focused on the glial fibrillary acidic protein promoter, which is a cognate marker of astrocytes that is expressed by enteric glia and non-myelinating Schwann cells. We describe the morphology of non-myelinating Schwann cells in the lung and verify that they express glial fibrillary acidic protein and S100, a classic glial marker. Furthermore, we characterize the relationship of non-myelinating Schwann cells to pulmonary nerves. Finally, we report tools for studying their function, including a commercially available transgenic mouse line.

## Introduction

In the lung, over 90% of all nerves are unmyelinated (Jammes et al., 1982) and are associated with non-myelinating Schwann cells, also called Remak cells. Non-myelinating Schwann cells in the lung were first described in 1957 (Agostoni et al., 1957), and electron microscopy studies of lung innervation consistently demonstrate a close association between these cells and the axons of pulmonary nerves (Cook and King, 1970; Fillenz, 1970; Hung et al., 1972; Fox et al., 1980; Jammes et al., 1982; Pack et al., 1984; Sparrow et al., 1999).

Autonomic pulmonary nerves regulate airway function and consist of cholinergic parasympathetic, adrenergic sympathetic, and non-adrenergic, non-cholinergic (NANC) branches (Downing and Lee, 1980; Nadel and Barnes, 1984; Belvisi, 2002). Preganglionic autonomic fibers are myelinated, while the postganglionic portion is unmyelinated. As with other visceral organs, parasympathetic ganglia are found within the lung and sympathetic ganglia are extrapulmonary (Purves and Lichtman, 1978; Loewy, 1981; Strack et al., 2002; Hanani, 2010).

The autonomic nervous system regulates a variety of pulmonary functions such as airway diameter via bronchial smooth muscle and vascular tone, mucus secretion, and chemosensation of gases (Undem and Nassenstein, 2009; Kc and Martin, 2010). The non-adrenergic, non-cholinergic system directly innervates bronchial smooth muscle and has both excitatory and inhibitory effects, which result in bronchoconstriction and bronchodilation, respectively (Stretton, 1991; Belvisi, 2002). More recently, it has been recognized that the pulmonary nerves also play a role in regulation of the inflammatory response. Inflammatory cells express nicotinic and muscarinic cholinergic, and α and β adrenergic receptors and the release of autonomic neurotransmitters can have pro- or anti-inflammatory effects depending on which receptors are stimulated (Verhein et al., 2009). Sympathetic nerve activity has been implicated in poststroke or traumatic brain injury immunosuppression and subsequent pneumonia (Prass et al., 2003, 2006; Sykora et al., 2015). The mechanisms behind neural regulation of the inflammatory response in the lung and possible participation by non-myelinating Schwann cells have not been elucidated.

However, it is likely that non-myelinating Schwann cells are important participants in pulmonary neural responses. In the gastrointestinal (GI) tract, enteric glia have been shown to play important roles in the formation and maintenance of the intestinal epithelial barrier, neurotransmission and immune response, and their dysfunction has been linked to inflammatory GI pathology (Bush et al., 1998; Cabarrocas et al., 2003; Gulbransen and Sharkey, 2012; Neunlist et al., 2012). There are several types of enteric glia, of which the intramuscular glia are most similar to non-myelinating Schwann cells in morphology and function (Gulbransen and Sharkey, 2012). All enteric glial types originate from a common population of neural crest progenitor cells that migrate to the GI tract during development and differentiate into either neurons or glia (Laranjeira et al., 2011).

Similarly, in the lung, both myelinating and non-myelinating Schwann cells arise from neural crest precursors that migrate from the esophagus into the mouse lung at embryonic day 10.5, accompanying the progression of peripheral nerves (Deal et al., 2006). In the developing human airways, a network of ganglia and nerve trunks accompanied by Schwann cells can be found by the middle of the first trimester (Sparrow et al., 1999). The calcium-binding protein S100, a classic glial marker, has been used to describe glia of the developing human respiratory tract (Sparrow et al., 1999) and adult lungs of several other mammals (Sheppard et al., 1983). However, very little is known about the anatomy, other cell-specific markers, or the function of non-myelinating Schwann cells in the lung, and there are no known transgenic mouse tools to study these cells *in vivo*.

The primary goal of this study was, therefore, to investigate the relationship of non-myelinating Schwann cells to pulmonary nerves in the airways and vasculature and to establish whether existing transgenic mouse tools would be useful for studying their function. We focused on the glial fibrillary acidic protein (GFAP) promoter, which is a cognate marker of astrocytes and expressed in enteric glial and non-myelinating Schwann cells (Jessen and Mirsky, 1985; Ruhl et al., 2004; Gulbransen and Sharkey, 2012). We first screened established, commercially available transgenic mouse lines that express green fluorescent protein (GFP) in the lung under the control of GFAP promoters and then used the mice to verify expression of the glial marker S100 and to describe the morphology, anatomic distribution, and association of glial cells with pulmonary nerves.

## Materials and Methods

### Animals

All animal procedures were performed in accordance with the protocol approved by the Institutional Animal Care and Use Committee at Stanford University. Six to 10-week-old mice on a C57BL/6J background purchased from The Jackson Laboratory, Bar Harbor, ME, were utilized for all studies: wild-type C57BL/6J mice (JAX#00664), GFAP-Cre (JAX#12886), ChAT-Cre (JAX#6410), Rosa26 eGFP (JAX#4077), Rosa26 Confetti (JAX#13731), and Rosa26 TDT (JAX#7914).

### Perfusion and Tissue Preparation

Mice were heavily anesthetized with chloral hydrate and terminally perfused through the right ventricle with 10–20 ml of cold 0.9% saline containing heparin (10 units/ml) until the lungs were completely clear of blood. Lungs were collected and drop fixed in 4% paraformaldehyde in phosphate buffer for 24 hr, transferred to 30% sucrose in phosphate buffer for 3–7 days, and 40 µm sequential coronal sections obtained using a freezing sliding microtome (Microm HM430).

### Immunohistochemistry

Immunohistochemistry was routinely performed on every 12th section so that all lung areas would be equally represented. Diaminobenzidine tetrahydrochloride (DAB) immunohistochemistry was performed. Free-floating sections were rinsed in Tris-buffered saline (TBS) and incubated with 2% hydrogen peroxide in 0.1% Triton X-100 for 1 hr at room temperature, then rinsed with TBS. Sections were blocked with 5% serum for 1 hr then incubated with primary antibodies (Table 1) diluted in 0.1% Triton X-100 and 3% serum overnight at room temperature. The following day, the sections were rinsed with TBS and incubated with a biotinylated secondary antibody (Table 2) for 1 hr. After rinsing with TBS, sections were incubated in avidin–biotin peroxidase complex (Vectastain Elite ABC kit, Vector Laboratories) for 1 hr, rinsed with TBS and a final wash with 0.1 M Tris and visualized with DAB for 1 to 2 min.

**Table 1.** Primary Antibodies Used for Immunohistochemistry.

Antigen	Description of immunogen	Supplier or catalog number	Species	Dilution
GFAP	GFAP purified from bovine spinal cord	Dako N Z0334	Rabbit polyclonal	1:4,000
GFAP*	Enriched bovine glial filaments	Invitrogen 13-0300	Rat monoclonal	1:200
GFAP*	Full length bovine native protein	Abcam ab4674	Chicken polyclonal	1:200
GFP	Recombinant GFP containing a 6-his tag	Millipore ab16901	Chicken polyclonal	1:200
MBP	Full length bovine protein	Abcam ab7349	Rat monoclonal	1:200
PECAM1 (CD31)	129/Sv mouse-derived endothelioma cell line tEnd.1	BD Biosciences 550274	Rat monoclonal	1:300
PGP9.5	Human PGP9.5 protein purified from pathogen-free human brain	UltraClone Limited RA95101	Rabbit poyclonal	1:500
S100	S100 purified from bovine brain	Dako Z0311	Rabbit polyclonal	1:1,000
αSMA	N-terminal synthetic decapeptide of α-smooth muscle chain	Sigma C6198 Cy3 conjugated	Mouse monoclonal	1:200
TH	Denatured TH from rat pheochromocytoma	Millipore AB152	Rabbit polyclonal	1:1,000

*Note*. GFAP = glial fibrillary acidic protein; GFP = green fluorescent protein; MBP = myelin basic protein; PECAM1 = platelet endothelial cell adhesion molecule-1; PGP9.5 = protein gene product 9.5; αSMA = α-smooth muscle actin; TH = tyrosine hydroxylase.

* Antibodies that stained weakly in lung, but showed strong, specific staining in brain.

**Table 2.** Secondary Antibodies Used for Immunohistochemistry.

Secondary antibody	Conjugated fluorophore	Supplier or catalog number	Dilution
Donkey anti-chicken IgY polyclonal	Cy 3	Jackson ImmunoResearch #703-165-155	1:200
Donkey anti-chicken IgY polyclonal	FITC	Jackson ImmunoResearch #703-096-155	1:200
Donkey anti-rabbit polyclonal	Alexa Fluor 555	Life Technologies #A-31572	1:200
Goat anti-rabbit polyclonal	Biotin	Vector Laboratories #BA-1000	1:200
Donkey anti-rabbit polyclonal	Alexa Fluor 647	Life Technologies #A-31573	1:200
Donkey anti-rat polyclonal	Cy3	Jackson Immunoresearch #712-165-153	1:200

### Immunofluorescence

Free-floating sections were rinsed with TBS and blocked for 1 hr in 5% serum from the species in which the secondary antibody was raised diluted in 0.1% Triton X-100. Sections were incubated overnight at room temperature in primary antibodies diluted in 0.1% Triton X-100 and 3% serum according to the concentrations in Table 1. The next day, sections were rinsed with TBS. Fluorescent secondary antibodies (Table 2) were centrifuged before use at 13,000 rpm for 10 min and then diluted 1:200 in 0.1% Triton X-100 and 3% serum. Sections were incubated in the appropriate secondary antibodies at room temperature for 4 to 6 hr, rinsed in TBS, wet mounted with Vectashield hard set mounting media with 4'6-diamidino-2-phenylindole (Vector Labs) and coverslipped.

### Image Acquisition and Analysis

Images of DAB-stained sections were taken using a Zeiss Axio Imager M1 with charge-coupled device camera using a 20× objective. Immunofluorescent sections were imaged using 40×, 1.15 numerical aperture and 63×, 1.30 numerical aperture oil objectives on a Leica TCS SPE confocal microscope using Leica Application Suite Advanced Fluorescence software. Z-stack images of fluorescent lung sections were acquired by sequential scanning every 1 µm and taken at a 2,048 × 2,048 pixel size, with a line average of 2 to reduce noise. Stacks were reconstructed using FIJI (NIH), and Photoshop (Adobe) software was used to change brightness and contrast of the images.

To quantify double labeling, we used an unbiased approach. For example, to examine whether GFP expression in GFAPcre-GFP mice was present in GFAP + cells, lung sections from three animals were immunostained for GFAP with a red (Alexafluor 555) secondary. When a GFP-expressing cell was identified in the green channel, the channel was switched to red to determine whether it also stained for GFAP. Conversely, to evaluate which percent of GFAP immunostained cells express GFP, GFAP + cells were identified in the red channel then the channel switched to green to score GFP expression. At least 100 cells from each mouse were counted for each of these questions.

### Antibody Characterization

Several antibodies against GFAP, an intracytoplasmic filamentous protein that forms part of the cytoskeleton of glial cells, were used. The rabbit polyclonal antibody produced by Dako has been used extensively to study glial cells both within the central nervous system (CNS) and in the periphery (Middeldorp and Hol, 2011; Yamazaki et al., 2011; Zamanian et al., 2012; Voss et al., 2013; Forrest et al., 2014). We have tested this antibody by Western blots of brain and lung homogenates; in both tissues, we find a specific band of 50 kDa, which is the appropriate size for GFAP. We have used this antibody for DAB and immunofluorescence in both brain (Cekanaviciute et al., 2014) and lung and find brightly stained cells that are of astrocyte morphology in brain and what would be expected of the morphology and distribution for non-myelinating Schwann cells in the lung. Other GFAP antibodies were tested (made in rat and chicken, Table 1), and although they stained strongly and specifically for astrocytes in the brain staining in the lung appeared to be specific but was extremely weak.

The Millipore anti-GFP antibody is made against highly purified native GFP from *Aequorea victoria* and reacts with both native and recombinant GFP sources. The manufacturer reports that the antibody binds to a specific 30-kDa band in Western blot of lysates from *Escherichia coli* expressing GFP. No band is seen in lysates from *E. coli* that do not express GFP. In immunocytochemistry, GFP is detected in cells transfected with a plasmid directing expression of GFP or GFP-fusion protein, cells that do not express GFP exhibit no detectable staining. In our laboratory, we have verified the specificity of this antibody through DAB immunostaining of lung and brain tissues from mice that express GFP under a GFAP promoter. Additionally, we have ensured that the staining is limited to GFP-expressing cells by using the GFP antibody with a 555 conjugated secondary.

Myelin basic protein (MBP) is one of the most abundant protein components of myelin both in the CNS and peripheral nervous systems (PNS). This Abcam antibody has been widely used as an oligodendrocyte and myelinating Schwann cell marker (Yu et al., 2009; Latimer et al., 2011; Fricker et al., 2013). The manufacturer has tested this antibody through immunohistochemistry of paraformaldehyde fixed frozen spinal cord sections, paraffin embedded brain sections, in which appropriate myelin staining was observed. On Western blots, two bands of 19 and 26 kDa represent MBP isoforms. In our lung immunostains, we observe sparse but present labeling of structures of appropriate morphology for myelin sheathes. In double stains, MBP-labeled structures can be seen surrounding nerves.

Platelet endothelial cell adhesion molecule-1, also known as CD31, reacts with adult and embryonic endothelial cells. The manufacturer has tested the antibody with immunohistochemical staining of zinc-paraffin sections of mouse spleen, lung, heart, and thymus and reports that it stains endothelial cells on small and large blood vessels. In our hands, this antibody shows widespread staining in lung parenchyma and larger blood vessels appropriate to vascular endothelium. Additionally, this antibody does not stain airway endothelial cells.

Protein gene product 9.5 (PGP9.5) is widely used as a pan-neuronal and neuroepithelial cell marker in the PNS, including the lung (Lauweryns and Van Ranst, 1988; Veres et al., 2007). The antibody labels neuronal cell bodies and axons and neuroendocrine cells in the CNS and PNS. *In vivo* substrates of PGP9.5 are largely unknown (Day and Thompson, 2010). According to the manufacturer, anti-human PGP9.5 is particularly suitable for detecting small nerve fibers in peripheral tissues. We observe specific staining morphologically appropriate for nerves and neuroepithelial bodies in the lung that have the correct anatomic distribution.

α-Smooth muscle actin specifically labels the α-smooth muscle isoform of actin and does not react with other major actin isoforms present in fibroblasts or epithelial cells, striated muscle, myocardium, or gamma-smooth muscle isoform. This antibody labels vascular and visceral smooth muscle cells in adult and embryonic tissues. α-Smooth muscle actin is a well-characterized marker of myofibroblasts used to immunolabel smooth muscle in lung (Cho et al., 2004; Lembrechts et al., 2011; Forrest et al., 2014) and other peripheral tissues (Arnold et al., 2013; Voss et al., 2013). Western blot validation by the manufacturer produces a specific 42-kDa band. In our lung stains, this antibody labels smooth muscle in the trachea and large bronchi, which results in a striated appearance that is appropriate for airway smooth muscle. Smooth muscle cells in blood vessel walls are also labeled.

Calcium-binding proteins, such as S100, are expressed in many cell types. S100B is most abundant in glial cells of the CNS and PNS but is also present in other cell types, including a subpopulation of neurons. The S100 antibody from Dako is widely used in the literature to immunostain peripheral glial cells (Young et al., 2002; D'Antonio, 2006; Voss et al., 2013; Kabouridis et al., 2015). According to the manufacturer, the S100 antibody used was solid-phase absorbed with human plasma and bovine serum proteins and on Western blot of purified human recombinant S100 proteins, this antibody labels S100B strongly, S100A1 weakly, and S100A6 very weakly. No reaction seen with other S100A proteins tested, including S100A4.

The Millipore antibody for tyrosine hydroxylase (TH) is widely used to study dopaminergic and adrenergic neurons in the CNS (Forrest et al., 2014). TH is the first rate-limiting enzyme involved in the synthesis of the catecholamines dopamine and noradrenaline from tyrosine. The manufacturer has tested the antibody through Western blot, which shows a specific 62-kDa band. In our hands, we observe characteristic immunostaining of sympathetic nerves that is limited to vesicles and, therefore, confers a beaded appearance. In lung, immunostaining is restricted to areas where sympathetic nerves are expected to be, surrounding airways and in blood vessel walls.

## Results

### Cells in the Mouse Lung Express Glial-Specific Markers S100 and GFAP

**Figure 1.**
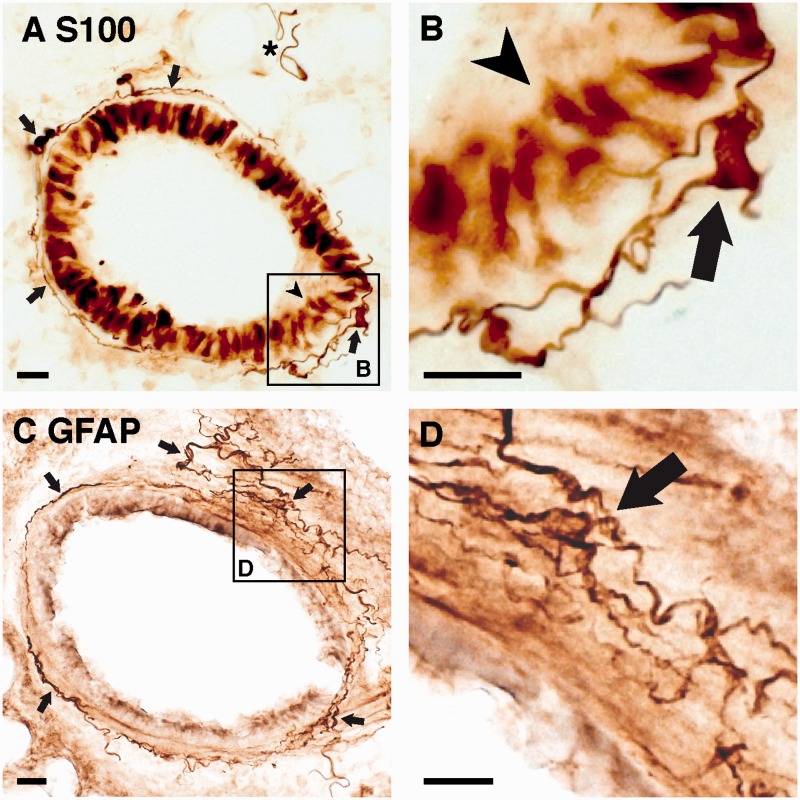
Glial markers S100 and GFAP immunostain elongated cells that surround bronchi in the mouse lung. (A) Representative photomicrograph of S100 immunostaining around a bronchus demonstrates elongated cells around the airway (arrows) and airway epithelial cell staining (arrowhead). Typical appearance of smoother and more serpentine cells (asterisk) that are associated with vascular structures adjacent to the airways. (B) Enlargement of the box in (A) demonstrating the morphology of S100 immunostained cells around the bronchus (arrow) and airway epithelial cell staining (arrowhead). (C) Representative photomicrograph of GFAP immunostaining in the lung also demonstrates elongated cells around airways (arrows). (D) Enlargement of the box in C demonstrating the morphology of GFAP immunostained cells around the bronchus (arrow). Scale bars, 20 µm.

We began our characterization of Schwann cells in the lung by immunostaining lung sections from wild-type C57BL/6J mice for two glial-specific markers. The calcium-binding protein S100B has been reported to be a marker for peripheral non-myelinating glia (Cocchia and Michetti, 1981; Sheppard et al., 1983; Stoll et al., 1989; Cabarrocas et al., 2003). We immunostained lung sections with an antibody that strongly labels S100B but exhibits minor cross-reactivity to S100A (S100 antibody). We found that there were indeed prominent, nucleated, elongated cells that were most notable around medium to larger airways and vascular structures (Figure 1(A) and (B), arrows), as would be expected for glia that accompany pulmonary nerves. We noted that some of these elongated cells exhibited a distinctly smoother, more serpentine appearance (Figure 1(A), asterisk). However, we also consistently observed immunostaining in some bronchial epithelial cells (Figure 1(A) and (B), arrowhead).

We next immunostained against GFAP using a polyclonal rabbit antibody that labels all GFAP isoforms (Clairembault et al., 2014). GFAP is a type III intermediate filament protein (Steinert and Roop, 1988) that is a cognate marker of astrocytes in the brain and has also been reported in peripheral non-myelinating Schwann cells and enteric glia (Jessen and Mirsky, 1985; Gulbransen and Sharkey, 2012). We observed GFAP-immunoreactive cells that were of similar morphology and in a similar location to the cells identified by S100 immunohistochemistry (Figure 1(C) and (D), arrows). In both immunostains, these cells form an intricate network that surrounds large airways. The distribution is very dense around the primary bronchi and becomes progressively less dense in more distal airways. We quantified the presence of GFAP-expressing cells by counting 100 bronchi that were over 100 µm in diameter and cut into a cross-sectional plane in each of three mice. All bronchi counted had GFAP-expressing cells located just outside the basal lamina and partially or completely surrounding the airways.

Colocalization of GFAP and S100 could not be confirmed via double-immunostaining because both antibodies are made in rabbit. Interestingly several other anti-GFAP antibodies, a monoclonal rat, and a polyclonal chicken antibody (Table 1), that in our hands specifically and strongly label brain astrocytes, produced extremely weak immunostaining when applied to lung tissue.

### GFAP Transgenic Mice Drive GFP Expression in GFAP and S100 Immunostained Cells

Transgenic mouse lines that express specifically in GFAP and S100 positive cells were, therefore, necessary to further characterize these cells immunohistochemically and also to study their function in the future. We turned to commercially available transgenic mouse tools to determine if we could use a mouse line containing a GFAP promoter to drive expression in peripheral non-myelinating Schwann cells. We evaluated lungs from four mice lines designed to express GFP in GFAP-expressing cells (Table 3). For each mouse line, we first investigated if GFP was present by DAB immunostaining of the lung and if it was present, whether it exhibited the anatomic location and morphology expected based on GFAP and S100 immunostaining. Next, in order to determine whether GFP expression was limited to cells expressing GFAP, we performed a fluorescent double stain.

**Table 3.** Evaluation of Mouse Lines That Express Green Fluorescent Protein (GFP) Under a Glial Fibrillary Acidic Protein (GFAP) Promoter.

Mouse line	Crossed with	Model	GFAP promoter	DAB stain vs GFP	GFP or GFAP colocalization	Original reference	Source
FVB/NJ-GFAP-GFP	None	Transgenic	2.2-kb human	Present	GFP not detectable with fluorescent stain	Zhuo et al. (1997)	JAX #3257
C57BL/6 J-GFAP-cre/ERT2	C57BL/6J-Rosa 26 eGFP	Transgenic	2.2-kb human	Present	GFP staining did not colocalize with GFAP	Ganat et al. (2006)	JAX #12849
C57BL/6J-GFAP-GFP Pleiades	None	Knock-in	2.2-kb human	None	None	Portales-Casamar et al. (2010)	MMRRC #32918
C57BL/6J-GFAP-Cre	C57BL/6J-Rosa 26 eGFP	Transgenic	15-kb mouse	Present	GFP staining colocalized with GFAP	Garcia et al. (2004)	JAX #12886

*Note*. GFAP = glial fibrillary acidic protein; DAB = diaminobenzidine tetrahydrochloride; GFP = green fluorescent protein.

The first three lines we evaluated did not exhibit appropriate GFP expression. In FVB/NJ-GFAP-GFP mice, GFP could be detected through DAB staining and appeared to be in the correct anatomic location and morphology for GFAP positive cells. However, when we used fluorescent antibodies, we did not observe GFP immunostaining. Next, we characterized GFP expression in lungs of C57BL/6J-GFAP-cre/ERT2 mice crossed with a C57BL/6J-Rosa 26 eGFP reporter line. Mice were dosed with 300 mg/kg of tamoxifen dissolved in corn oil orally for 5 consecutive days and evaluated 4 days later. We observed extensive nonspecific GFP immunostaining in the lung, and in addition GFP was also not present in most cells that immunostained for GFAP. We then tested the C57BL/6J-GFAP-GFP Pleiades transgenic mouse, but no GFP expression was found in lung.

**Figure 2.**
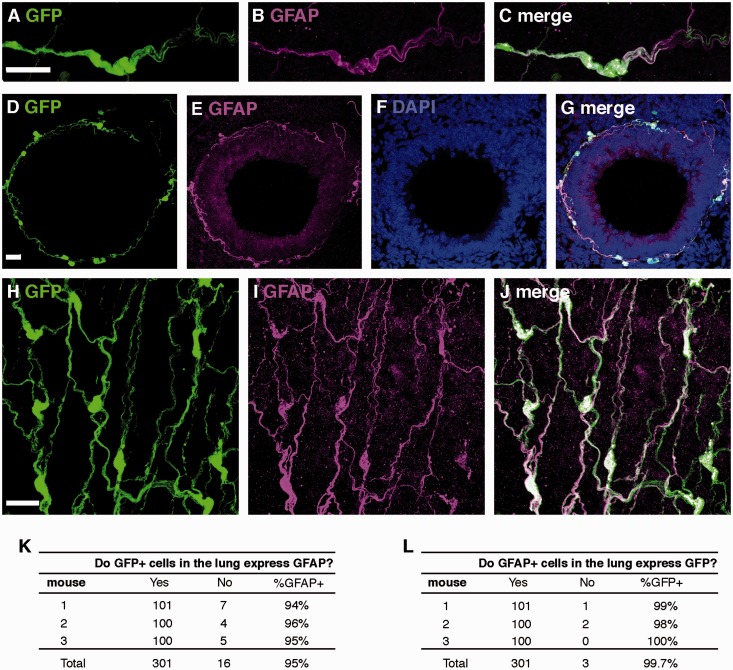
GFP expression and GFAP immunostaining in lungs of GFAPcre-GFP transgenic mice. (A–C) Representative colocalization of GFP and GFAP-immunostained cells in a multiaxon bundle. (D–G) Representative colocalization of GFP and GFAP immunostaining in networks of cells surrounding airways, in both cross-sectional (D–G) and longitudinal (H–J) planes. (K and L) Quantification of GFP expression and GFAP immunostaining in GFAP-cre-GFP mice. Scale bars, 20 µm.

The last strain we evaluated was a C57BL/6J-GFAP-Cre crossed with C57BL/6J-Rosa26 eGFP reporter mice, which we will abbreviate as GFAPcre-GFP mice here. GFAPcre-GFP mice exhibited GFP expression in the expected morphology and location for non-myelinating Schwann cells and excellent colocalization with GFAP immunostaining (Figure 2). To formally test concordance, we counted 100 cells per mouse and found that 95% of cells that express GFP also immunolabeled for GFAP (Figure 2(K)). In addition, 98% of cells that immunostained for GFAP coexpressed GFP (Figure 2(L)).

**Figure 3.**
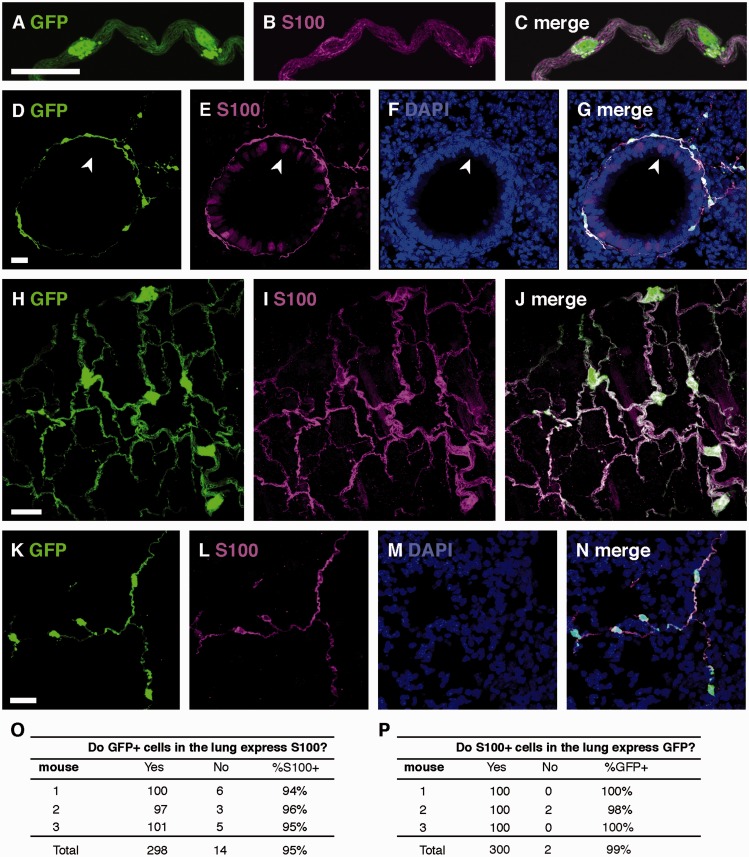
GFP and S100 immunostaining in lungs of GFAPcre-GFP transgenic mice. (A–C) Representative colocalization of GFP and S100-immunostained cells in a multiaxon bundle. (D–G) Representative colocalization of GFP and S100 immunostaining in networks of cells surrounding airways and epithelial cell staining (arrowheads) in cross-sectional (D–G) and longitudinal (H–J) planes. (K–N) GFP and S100 immunostaining in small airway cells. (O and P) Quantification of GFP expression and S100 immunostaining in GFAP-cre-GFP mice. Scale bars, 20 µm.

To verify that the morphologically similar cells that we had immunostained in wild-type mouse lungs for GFAP and S100 (Figure 1) were the same cells, we evaluated lungs from GFAPcre-GFP mice with anti-S100 antibody (Figure 3). The GFAPcre-GFP mice exhibited close concordance of S100 immunostaining with GFP expression. The bronchial epithelial cells seen in the DAB stain for S100 were also detected in fluorescent stains, but they did not colocalize with GFP (Figure 3(D)–(G), arrowhead). GFP expression in the distal airways was sparse, and these cells did coimmunostain with S100 (Figure 3(K)–(N)). Ninety-five percent of GFP-labeled cells around airways colocalized with S100 (Figure 3(O)) and 99% of S100-immunostained cells also exhibited GFP immunostaining (Figure 3(P)). GFAP staining was not found in distal airways; however, GFAPcre-GFP mice expressed GFP, which coimmunostained with S100 and PGP9.5.

**Figure 4.**
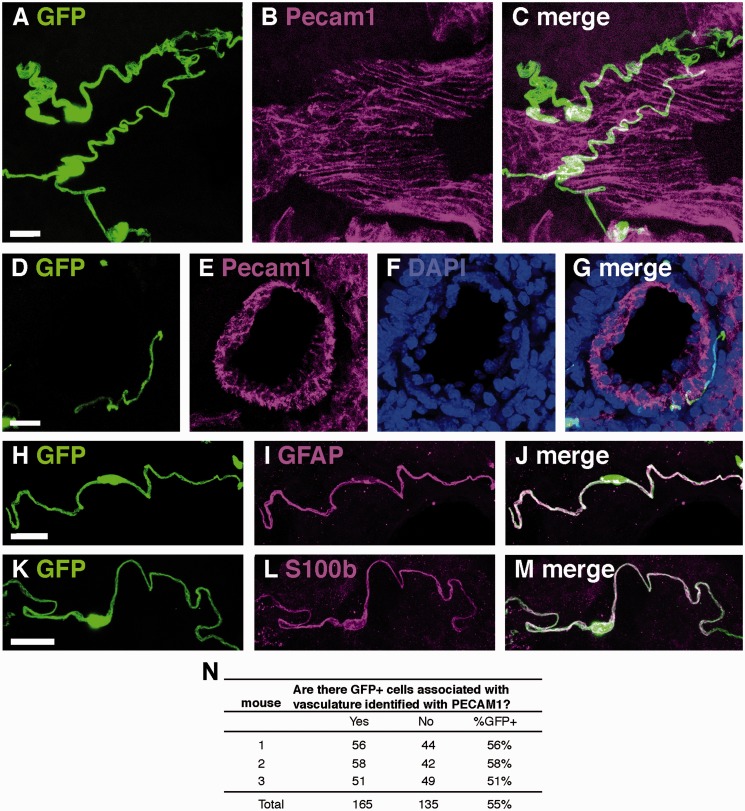
GFP and PECAM1 immunostaining in lungs of GFAPcre-GFP transgenic mice. (A–C) Representative image of GFP expressing cells associated with PECAM1 immunostained blood vessels longitudinally and (D–G) in cross-section. (H–J) Representative colocalization of GFP and GFAP immunostaining in vasculature associated cells. (K–M) Representative colocalization of GFP and S100 immunostaining in vasculature associated cells. (N) Quantification of GFP-expressing cells associated with pulmonary vasculature in GFAPcre-GFP mice. Scale bars, 20 µm.

We noted that the smooth, serpentine cells that immunostained for GFAP and S100 in DAB appeared to be associated with pulmonary blood vessels, which were identified by their characteristic morphology and anatomic position in relation to airways. We confirmed that these distinctly shaped cells were exclusively associated with blood vessels by immunostaining GFAPcre-GFP mouse lungs with the vascular endothelial marker platelet endothelial cell adhesion molecule-1 (PECAM1, Figure 4). As with the cells associated with airways, vasculature-associated GFP + cells coimmunolabeled with both GFAP (Figure 4(H)–(J)) and S100 (Figure 4(K)–(M)). In contrast to the airways, which almost always had GFP + cells surrounding them, only 55% medium to large blood vessels exhibited GFP-expressing cells (Figure 4(N)).

In GFAPcre-GFP mouse lungs, all of the cells associated with large airways and vasculature that express GFP also exhibit GFAP and S100 immunostaining. The cells that do not exhibit GFAP immunostaining but do express GFP are the small airway-associated cells that also costain for S100 (Figure 3(K)–(N)).

### GFAP-Expressing Cells in the Lung Are Associated With Pulmonary Nerves

**Figure 5.**
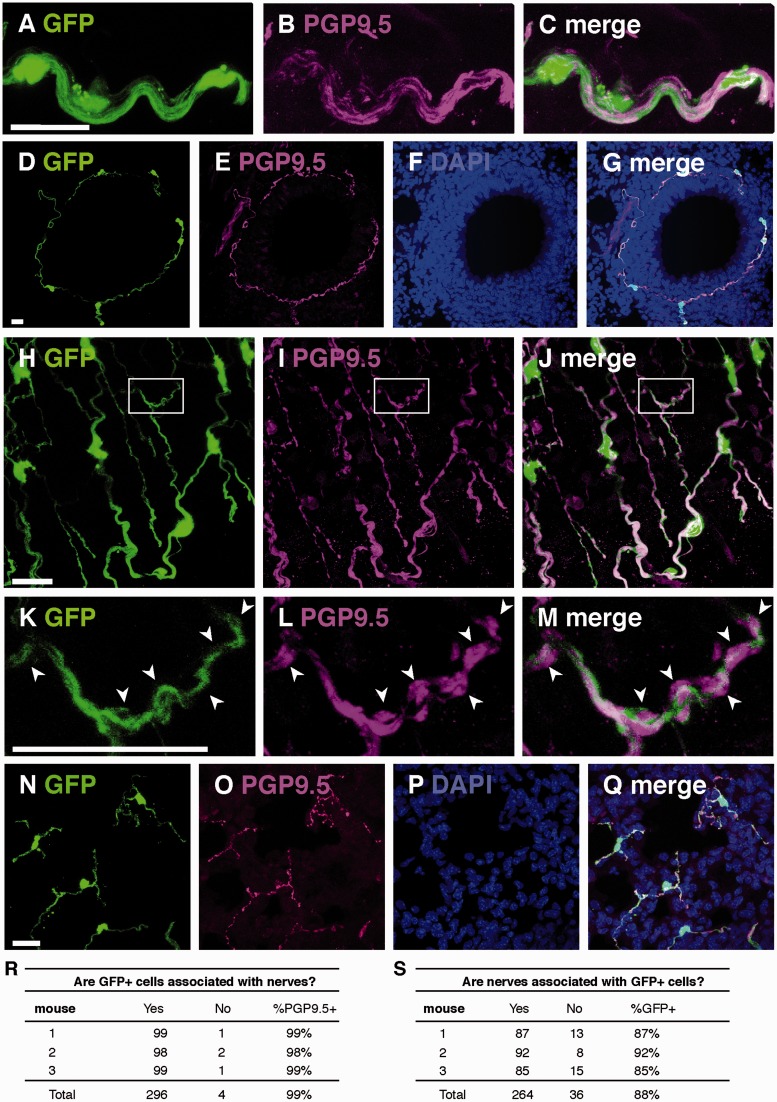
GFP and PGP9.5 immunostaining in lungs of GFAPcre-GFP transgenic mice. (A–C) Representative colocalization of GFP and PGP9.5-immunostained cells in a multiaxon bundle (D–G). Representative colocalization of GFP and PGP9.5 immunostaining in networks of cells surrounding airways in both cross-sectional (D–G) and longitudinal (H–J) planes. (K–M) Enlargement of the boxes in (H–J) demonstrating that fenestrations in non-myelinating Schwann cells colocalize with and axonal densities (arrowheads) in PGP9.5 immunostained nerves. (N–Q) GFP and PGP9.5 immunostaining in small airway cells. (R and S) Quantification of GFP-expressing cells associated with PGP9.5 immunostained nerves in GFAPcre-GFP mice. Scale bars, 20 µm.

An important characteristic of both myelinating and non-myelinating Schwann cells is close association with neurons or nerves. Peripheral nerves, including those in the lung (Lauweryns and Van Ranst, 1988), immunostain with the marker PGP9.5. Dual labeling of GFP and PGP9.5 in GFAPcre-GFP mice showed that GFP-expressing cells were closely associated with PGP9.5 immunostained nerves (Figure 5). PGP9.5-immunostained cells consistently exhibited axonal densities that are larger and more intensely stained than the remainder of the axon (Figure 5(K)–(M), arrowheads), and the glial cell sheath appears to have fenestrations that coincide with these densities. Of note, the GFP + cells in the distal airways that costain for S100 (Figure 3(K)–(N)) are also associated with PGP9.5 labeled nerves (Figure 5(N)–(Q)). Almost all GFP-expressing cells were associated with PGP9.5 immunostained nerves (Figure 5(R)), and 12% of nerves did not associate with GFP, which may correspond to myelinated nerves or naked nerve terminals (Figure 5(S)).

**Figure 6.**
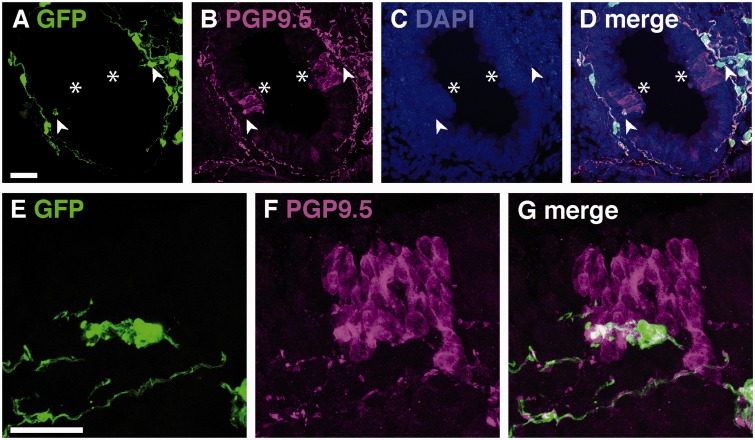
GFP and PGP9.5 immunostaining neuroepithelial bodies of GFAPcre-GFP transgenic mice. (A–D) Representative image of GFP-expressing cells that contact the basal aspect (arrowheads) of PGP9.5 immunostained neuroepithelial bodies (asterisks). (E–G) A magnified view of GFP-expressing cells contacting the basal aspect of a PGP9.5 immunostained neuroepithelial body. Scale bars, 20 µm.

PGP9.5 also immunostains neuroepithelial bodies which we identified by their characteristic morphology and location within the airway lumen (Figure 6). Neuroepithelial bodies are clusters of specialized neuroendocrine cells, a distinct population of epithelial cells that have endocrine secretory functions (Van Lommel et al., 1999) and are supplied by a variety of nerve types (Brouns et al., 2008). Neuroepithelial bodies did not express GFP and were not ensheathed by GFP-expressing cells. However, GFP + non-myelinating Schwann cells appeared to be in contact with the basal aspect of neuroepithelial bodies.

### Non-Myelinating Schwann Cells Are Closely Associated With Sympathetic Nerves

**Figure 7.**
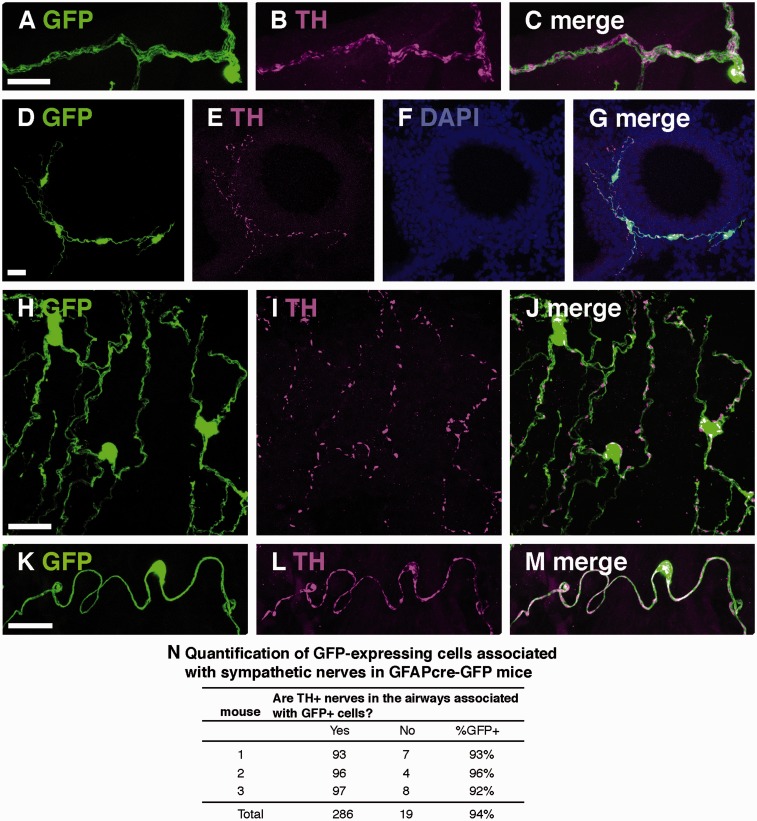
GFP and TH immunostaining in lungs of GFAPcre-GFP transgenic mice. (A–C) Representative co-localization of GFP and TH-immunostained cells in a multiaxon bundle. (D–G) Representative colocalization of GFP and TH immunostaining in networks of cells surrounding airways in both cross-sectional (D–G) and longitudinal (H–J) planes. (K–M) Blood-vessel associated non-myelinating Schwann cells (GFP) with TH immunostaining. (N) Quantification of GFP-expressing cells associated with sympathetic nerves in GFAPcre-GFP mice. Scale bars, 20 µm.

We also examined colocalization of GFP in GFAPcre-GFP mice with sympathetic nerves (Figure 7), which innervate both the airways and vasculature of the mouse lung. We used TH to immunostain sympathetic nerves and their terminals. Sympathetic cell bodies are located outside of the lung; the superior cervical and stellate ganglia supply the trachea, and the stellate ganglion and thoracic ganglia 2 to 6 provide sympathetic innervation to the rest of the lung (Honjin, 1956; Kummer et al., 1992). GFP-expressing glia in the lung, on the other hand, do have nuclei, so non-myelinating Schwann cells could be identified by their nuclei despite their intimate association with sympathetic axons. Whenever a serpentine shaped, GFP-expressing cell was noted it was always associated with a vascular structure and was always positive for TH immunostaining (Figure 7(K)–(M)). In airways, TH + sympathetic fibers were nearly always associated with GFP + cells (94%, Figure 7(N)) and both as part of nerve bundles and single fibers. In contrast, sympathetic fibers associated with vascular structures were often found without an associated non-myelinating Schwann cell.

### Myelinated and Unmyelinated Autonomic Nerves in the Lung Travel in Large Nerve Bundles

**Figure 8.**
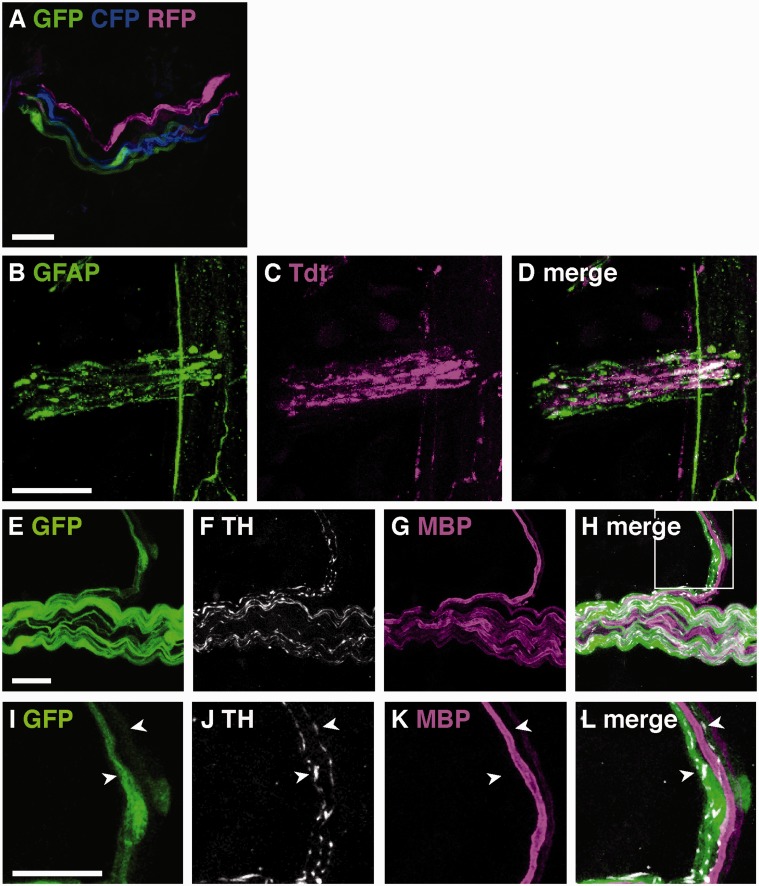
(A) GFP, CFP, and RFP expression in lungs of GFAPcre-Confetti transgenic mice. (B–D) GFAP immunostaining and TDT expression in lungs of ChATcre-TDT transgenic mice. Representative image of coimmunostaining cholinergic, parasympathetic multiaxon bundle. (E–H) Representative image of a multiaxon bundle stained for GFP, TH, and MBP in lungs of GFAPcre-GFP mice. (I–L) Enlargement of box in (H). TH immunostained sympathetic nerve fibers are ensheathed by GFP expressing cells in a bundle that also contains MBP immunostained structures that do not colocalize with either GFP or TH. Scale bars, 20 µm.

To distinguish one cell from the other and determine the extent and territory of individual glial cells, we crossed GFAP-cre mice to Rosa 26 Confetti mice, in which cre-driven recombination results in expression of yellow or green, red, or cyan fluorescent proteins in adjacent cells. We found that GFAP-expressing glia were present in these nerve bundles and that multiple glia line up longitudinally along each nerve fiber (Figure 8(A)).

Myelinated nerves in the lung are predominantly pre-ganglionic cholinergic parasympathetic nerves, but there are also some myelinated sensory nerves (Brouns et al., 2005). We observed prominent nerve bundles in the largest airways that immunostained for GFAP in parasympathetic nerve reporter mice (choline acetyl transferase (ChAT)-cre mice crossed with Rosa26-tandem dimer tomato (TDT) reporter line, Figure 8(B)–(D)). We observed close association of GFAP and thinner TDT positive fibers; however, thicker fibers that formed part of larger bundles did not clearly colocalize with GFAP, implying that myelinating Schwann cells do not consistently coexpress GFAP. Indeed, unmyelinated TH-expressing sympathetic nerves did clearly associate with GFP in GFAPcre-GFP mice but neither marker associated with MBP in multiaxon bundles (Figure 8(E)–(L)).

## Discussion

**Figure 9.**
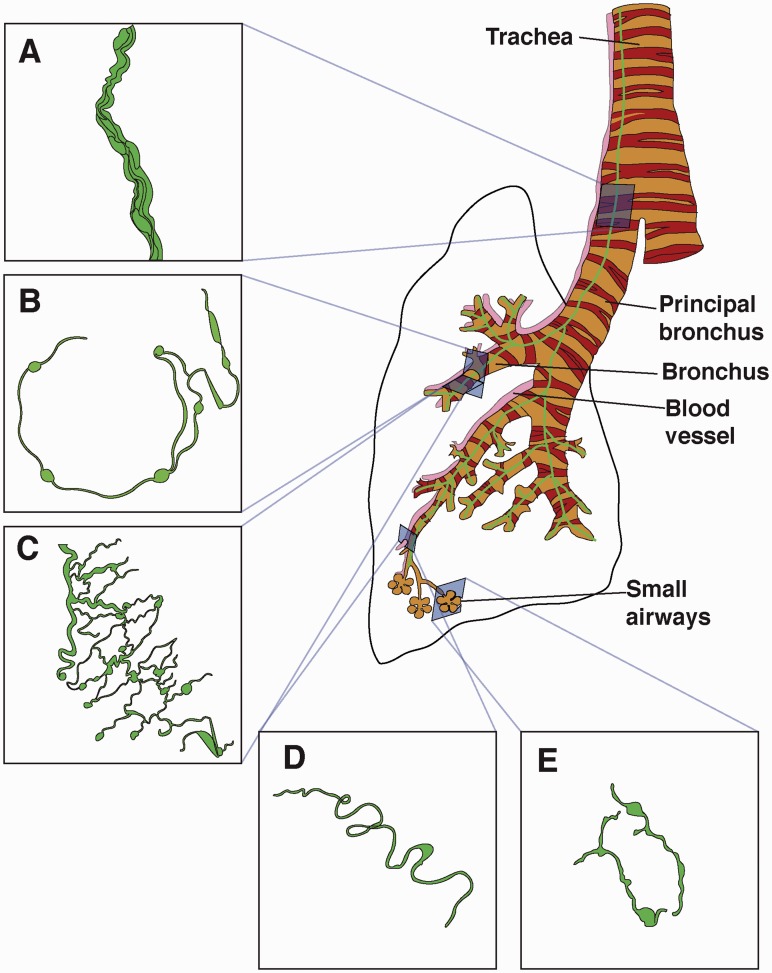
Diagram of a mouse lung showing the location of non-myelinating Schwann cells (A). Nerves travel down the lung in thick bundles of nerves that are ensheathed by glia. Glia-ensheathed nerves form a mesh that surround large and small bronchi and form an almost complete semicircle around the cross-sectional aspect of the airways (B) and appear as a network on the longitudinal aspect (C). Glia are also found ensheathing blood vessel nerves (D), although at a much lower density compared with airways. Blood vessel-associated glia have a smoother and more serpentine morphology compared with airway glia. In small airways, (E) GFAP immunostaining is not visible; however, we do find the presence of GFP in the small airways GFAP-GFP mice. Although the morphology of the GFP + cells found in small airways is different to that of larger airways or blood vessels, these cells do co-immunostain with other glial markers (S100) and nerve markers (PGP9.5).

In the present study, we describe GFAP-expressing non-myelinating Schwann cells in the lung. We validate a transgenic mouse line that drives expression of cre under a GFAP promoter and demonstrate that in GFAPcre-GFP mice, GFP is widely expressed but limited to non-myelinating Schwann cells that immunostain for GFAP and S100 and are associated with pulmonary nerves. Non-myelinating Schwann cells ensheathe pulmonary nerves that travel distally in large bundles (Figure 9(A)) along the bronchial tree. They also ensheathe the nerves that branch off from the bundles to form part of an intricate bronchial plexus, which can be seen in the cross-sectional plane surrounding bronchi (Figure 9(B)) and have a mesh-like appearance in the longitudinal plane (Figure 9(C)). Glia that are associated with the pulmonary vasculature also immunostain for S100 and GFAP are sparse and have a characteristic morphology that is smoother and more serpentine than airway-associated glial cells (Figure 9(D)). In small airways, GFAP immunostaining is not detectable, however, slender, elongated GFP-expressing cells also immunostain for S100 and are associated with nerves immunostained with PGP9.5 (Figure 9(E)). Non-myelinating Schwann cells are intimately associated with TH immunolabeled sympathetic nerves both in airways and blood vessels.

Beginning in the 1950s, electron microscopy studies revealed the fine structure of unmyelinated peripheral nerve fibers and Schwann cells (Hess and Lansing, 1953). Later research showed that the airways of mammals, including mice (Hung et al., 1972), rats (Jeffery and Reid, 1973), and humans (Fox et al., 1980) are supplied by unmyelinated nerve axons that are partially or completely surrounded by non-myelinating Schwann cells. Similar studies have explored the innervation of other visceral organs such as the liver (Yamada, 1965), kidney (Fazan et al., 2002), spleen (Felten and Olschowka, 1987), adrenal medulla (Coupland, 1965), and bone marrow (Yamazaki and Allen, 1990), among others, but Schwann cells are mentioned only in the context of nerve axons.

No unifying functions of glial cells in visceral organs are known, or if they are specialized by organ. In the bone marrow, non-myelinating Schwann cells are involved in maintenance of the hematopoietic stem cell niche (Yamazaki et al., 2011). In the lung, heart, intestine, bladder, and kidney non-myelinating Schwann cells express a specialized sodium channel and have been implicated to play a role in extracellular sodium level regulation (Watanabe et al., 2002). There is a growing interest in enteric glia as a component of the immune barrier in the gut (Bush et al., 1998; Cornet et al., 2001; Cabarrocas et al., 2003; Savidge et al., 2007; Gulbransen and Sharkey, 2012) that responds to sympathetic nerve stimulation (Gulbransen et al., 2010). Enteric glia differ from non-myelinating Schwann cells (Gershon and Rothman, 1991); however, they are the best characterized visceral glia and may have common functional pathways with other visceral glia.

The availability of this GFAP-cre transgenic mouse tool makes it possible to study the functions of pulmonary glia. We chose to evaluate GFAP expression in the lung because it is a marker of peripheral non-myelinating Schwann cells and enteric glia (Jessen et al., 1984; Gulbransen and Sharkey, 2012). Additionally, there are commercially available mice that express transgenes under GFAP promoters. However, few transgenic tools have been validated in non-myelinating Schwann cells. A transgenic line of S100B-GFP mice express GFP specifically in brain astrocytes (Vives et al., 2003); in the kidney, however, GFP is also expressed in cuboidal epithelial cells of Bowman's capsule (Darlot et al., 2008). Given that some bronchial epithelial cells immunostain for S100, it is possible that S100B-GFP mice also express GFP in those pulmonary cells. Some transgenic mouse lines have been created to study activity of specific gene functions of non-myelinating Schwann cells: one expresses a dominant negative ErbB2 receptor under the control a GFAP promoter preferentially in the PNS (sciatic nerve, Chen et al., 2003 and inner ear, Rio et al., 2001) and minimally in the CNS or GFAP-IkB-alpha-dn mice, in which glial NF-κB signaling is disrupted in the sciatic nerve-associated non-myelinating Schwann cells (Fu et al., 2010).

We establish here that GFAP-cre mice will be a useful tool to study non-myelinating Schwann cells in the lung. When crossed to Rosa26 GFP reporter mice, expression is remarkably faithful to GFAP immunohistochemistry and limited to non-myelinating Schwann cells identified with GFAP and S100 immunostaining. Only 5% of GFP expressing cells do not immunostain for GFAP or S100; however, their morphology and localization closely resembles those cells that immunostain for these glial markers. We may not have always observed GFP colocalizing with GFAP because GFP is particularly concentrated in the nuclei and decreases as it spreads out to the rest of the cell processes, while GFAP immunostaining tends to be stronger in the processes and less within the nucleus. Additionally, cells that immunostain for either GFAP or S100 overwhelmingly (99%) express GFP.

We did not see convincing evidence of GFAP expression in myelinating Schwann cells and propose here that it is an exclusive marker for non-myelinating Schwann cells in the lung. The large nerve bundles that are associated with large airways contain tightly packed myelinated and unmyelinated axons. Despite this, we never observed GFP clearly colocalized with MBP immunostaining in GFAPcre-GFP mice. In non-transgenic mice, we very occasionally observed some GFAP immunostaining on the cut edge of a bundle of myelinated nerves. This was also observed with other antibodies, and although we think is an artifact, myelinating Schwann cells have been reported to express GFAP when there is axonal injury (Stoll and Müller, 1999). Therefore, although we cannot exclude that some myelinating Schwann cells may express GFAP after injury, we conclude that in uninjured mice GFAP expression is confined to non-myelinating Schwann cells.

There are reports of GFAP-expressing lung cells that are not glial cells but are instead peribronchial fibroblasts, with Zhao and Burt (2007) attributing them to a “stellate cell” network (Hainfellner et al., 2001). In contrast, we report here that GFAP-expressing cells in the lung are non-myelinating Schwann cells that are associated with pulmonary nerves. We did not observe GFAP-expressing cells that were not associated with pulmonary nerves. The previous reports did not perform double immunofluorescence with nerve markers. The morphology of the GFAP immunostained cells reported in the lung by Zhao and Burt is similar to the GFAP immunostaining that we show here but with shorter processes. We speculate that this is because they used 10 µm paraffin embedded sections while we used 40 µm thick floating sections, and that in their slides, Schwann cell processes were transected. We do report here a population of cells within the inner lining of the airways that immunostain for S100, but not for GFAP or GFP. However, these cells are unlikely to be fibroblasts since their anatomy and morphology is very characteristic of airway epithelial cells. Finally, the S100 antibody that we used does not immunostain for S100A4, which is also known as fibroblast specific protein 1, a specific fibroblast marker.

Although GFAP expression has been reported in neural crest precursor cells (Johnson et al., 2009), cre recombinase did not appear to be expressed in neuronal axons in this GFAPcre-GFP mouse line. The resolution that we achieved with confocal microscopy was not sufficient to convincingly show Schwann cells and nerve axons as separate cells. However, electron microscopy images of non-myelinating Schwann cells in the lung clearly show that each axon is partially or completely surrounded by Schwann cell processes (Hung et al., 1972). Our immunostaining is thus consistent with this relationship between non-myelinating Schwann cells and axons. Additionally, sympathetic nerve axons have their cell bodies outside of the lung (Strack et al., 2002); therefore, the nuclei that are found associated with TH + nerves belong to glia, not nerves. We found that non-myelinating Schwann cells do not ensheathe neuroepithelial bodies; however, they do contact the basal aspect and are probably associated with capsaicin-sensitive, calcitonin gene-related peptide and substance P immunoreactive nerve fibers, which contact neuroepithelial bodies at their basal pole only (Brouns et al., 2005).

This is, to our knowledge, the first characterization of non-myelinating Schwann cells in the lung, including their basic morphology and anatomic distribution as well as immunohistochemical markers and relationships to lung innervation. We also identify a GFAP-cre mouse line that can be bred to floxed lines to disrupt genes or pathways of interest in non-myelinating Schwann cells in the lung.

## Summary Statement

We describe the morphology and anatomic distribution of pulmonary glia, non-myelinating Schwann cells that surround pulmonary autonomic nerves and validate a transgenic mouse tool to study the function of these cells.
